# The essential role of GATA transcription factors in adult murine prostate

**DOI:** 10.18632/oncotarget.10294

**Published:** 2016-06-25

**Authors:** Lijuan Xiao, Qin Feng, Zheng Zhang, Fen Wang, John P. Lydon, Michael M. Ittmann, Li Xin, Nicholas Mitsiades, Bin He

**Affiliations:** ^1^ Department of Medicine, Section of Hematology and Oncology, Baylor College of Medicine, Houston, TX, USA; ^2^ Department of Molecular and Cellular Biology, Baylor College of Medicine, Houston, TX, USA; ^3^ The Center for Cancer and Stem Cell Biology, Institute of Bioscience and Technology, Texas A&M Health Science Center, Houston, TX, USA; ^4^ Department of Pathology and Immunology, Baylor College of Medicine, Houston, TX, USA; ^5^ Michael E. DeBakey Veterans Affairs Medical Center, US Department of Veterans Affairs, Houston, TX, USA

**Keywords:** GATA2, GATA3, FoxA1, androgen receptor, prostate cancer

## Abstract

GATA transcription factors are essential in mammalian cell lineage determination and have a critical role in cancer development. In cultured prostate cancer cells, GATA2 coordinates with androgen receptor (AR) to regulate gene transcription. In the murine prostate, among six GATA members, GATA2 and GATA3 are expressed. Immunofluorescence staining revealed that both GATA factors predominantly localize in the nuclei of luminal epithelial cells. The pioneer factor FoxA1 is exclusively detected in the luminal cells, whereas AR is detected in both luminal and basal cells. Using genetic engineering, we generated prostate-specific GATA2 and GATA3 knockout (KO) mice. Ablation of single GATA gene had marginal effect on prostate morphology and AR target gene expression, likely due to their genetic compensation. Double KO mice exhibited PIN III to IV lesions, but decreased prostate to body weight ratio, altered AR target gene expression, and expansion of p63-positive basal cells. However, deletion of GATA2 and GATA3 did not reduce the mRNA or protein levels of AR or FoxA1, indicating that GATA factors are not required for AR or FoxA1 expression in adult prostate. Surprisingly, GATA2 and GATA3 exhibit minimal expression in the ventral prostatic (VP) lobe. In contrast, FoxA1 and AR expression levels in VP are at least as high as those in anterior prostatic (AP) and dorsal-lateral prostatic (DLP) lobes. Together, our results indicate that GATA2 and GATA3 are essential for adult murine prostate function and *in vivo* AR signaling, and the lack of the GATA factor expression in the VP suggests a fundamental difference between VP and other prostatic lobes.

## INTRODUCTION

Prostate is a compound tubule-alveolar secretory gland of the male reproductive system in mammals. It is located at the base of the bladder and surrounds the urethra [1]. The prostate secretes about 30% of the seminal fluid that nourishes and protects spermatozoa. The human prostate gland is walnut sized and can be classified into four “zones”, including peripheral zone, central zone, transition zone, and anterior fibro-muscular zone (or stroma). In humans, prostate cancer arises mainly in the peripheral zone, whereas benign prostatic hyperplasia (BPH) occurs only in transition zone [1]. Mouse models have been valuable tools for studying the physiology of human prostate diseases [2, 3]. Although there are significant similarities between human and mouse prostate glands, there are major anatomical differences between them. Murine prostate is divided into distinct lobes including, anterior prostatic (AP) lobe, dorsal-lateral prostatic (DLP) lobes, and ventral prostatic (VP) lobe [1, 4], while human prostate is encased into one gland by stromal, each lobe of murine prostate is separated and surrounded by fibrous and adipose connective tissue. Functionally murine DLP is most analogous to the peripheral zone of the human prostate [5], whereas AP most closely resemble the central zone [2, 5].

Androgen signaling is essential for the development and normal function of the prostate. Male androgen testosterone is converted to more potent androgen dihydrotestosterone (DHT) in the prostate by 5-alpha reductase (SRD5A2). When bound to DHT, AR binds to the enhancer and promoter regions of target genes, such as PSA and TMPRSS2, and activates their transcription. AR KO male mice do not develop the prostate, seminal vesicle, vas deferens, and epididymis [6, 7]. In addition, several other key transcription factors also play essential roles in prostate development. FoxA1 interacts with AR and functions as a potent pioneer factor for AR genomic binding [8–11]. FoxA1 KO mice do not develop mature luminal epithelial cells [12] and FoxA1 is required for the maintenance of epithelial cell differentiation status in adult mouse prostate [13]. NK3 homeobox 1 (Nkx3- 1) is an AR downstream target gene in the prostate and maintains the differentiation status of luminal epithelial cells [14]. Nkx3-1 null mice develop prostatic epithelial hyperplasia [15].

The GATA family of transcription factors determines the cell lineages in numerous tissues in species from Drosophila to mammals [16]. In mammals, there are six GATA family members, GATA1 to GATA6. Each GATA member contains two highly conserved zinc fingers of the C2H2 type. Carboxyl-terminal zinc finger is responsible for DNA binding, while NH_2_-terminal zinc finger is involved in co-regulator interaction. GATA1, 2, and 3 have key roles in hematopoietic cells, while GATA4, 5, and 6 function in lung, heart, muscle, gastrointestinal and neuronal development. Malfunction of GATA family transcription factors are linked to a variety of human diseases including cancer. Germline heterozygous mutation of GATA2 causes leukemia [17, 18]. GATA3 functions in T lymphocytes and is critical for Th2 cell differentiation [19]. GATA2 and GATA3 are also expressed in the endothelia and central nervous system. For instance, GATA3 is essential for murine mammary gland development [20, 21] and is one of the most frequently mutated genes in human breast cancer [22, 23], suggesting a tumor suppressor function in the mammary gland. GATA2 and GATA3 mRNAs are expressed in human and murine prostate [24]. In cultured prostate cancer cell lines, GATA2 has been shown to function as a pioneer factor for AR genomic binding and function [24–27], while surprisingly GATA3 is not expressed. However, the *in vivo* function of GATA transcription factors in normal prostate remains unknown. In this study, using genetically engineered mouse models, we investigated the physiological role of GATA family transcription factors in the adult murine prostate.

## RESULTS

### Expression of the GATA family of transcription factors in the murine prostate

It has been reported that among six members of the GATA family of transcription factors, GATA2 and GATA3 are expressed in both murine and human prostate [24]. In agreement with this observation, in the purified total prostate epithelial cells, based on the microarray-based mRNA levels, GATA2 and GATA3, but not other members, are abundantly expressed (Supplementary Figure 1A).

**Figure 1:**
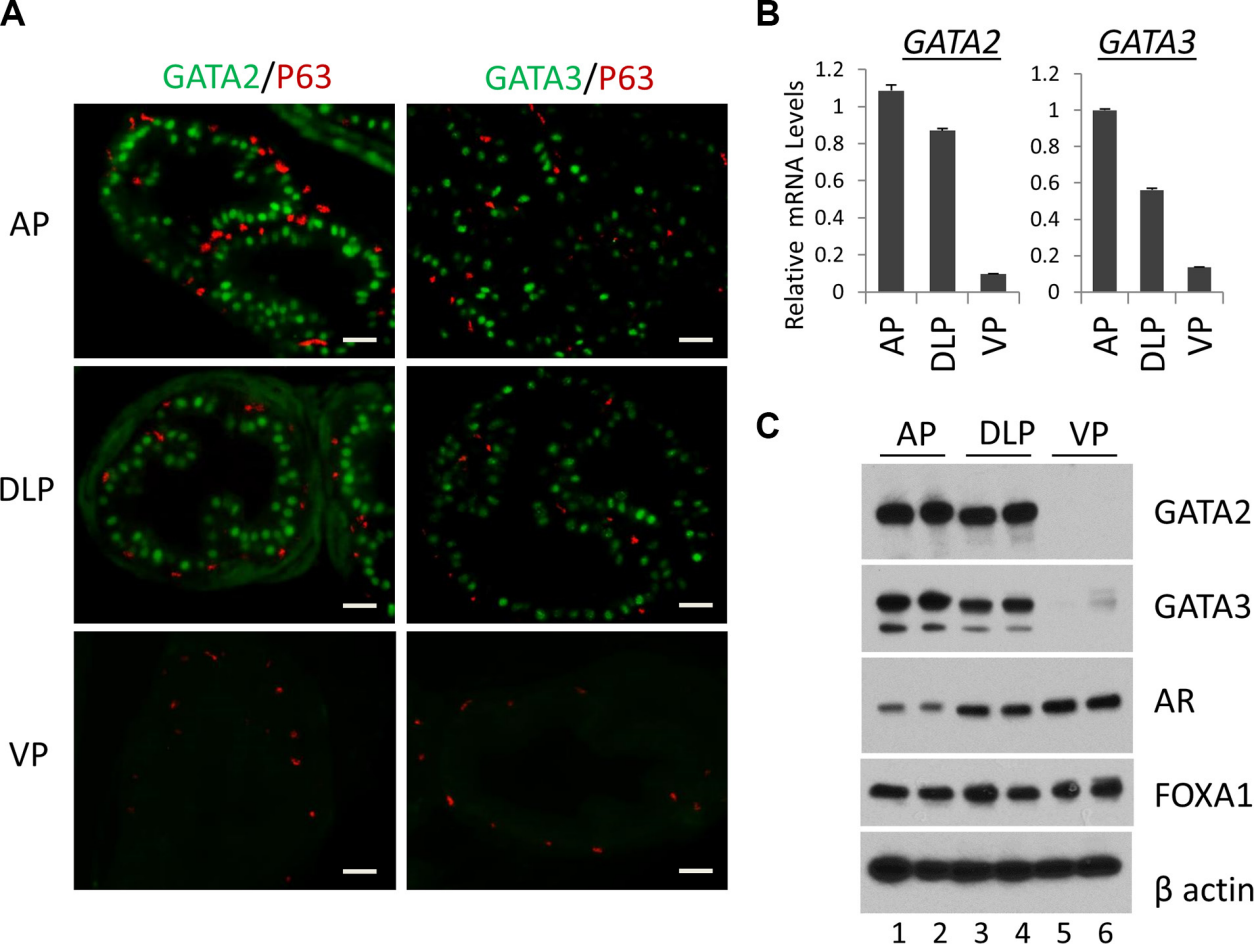
Expression of the GATA and other key transcription factors in WT murine prostate (**A**) Co-immunofluorescence staining of GATA2 (green), GATA3 (green) and p63 (red) in prostatic lobes. The scale bars, 20 μm in all panels. (**B**) mRNA levels of GATA2 and GATA3 in three prostatic lobes as determined by real-time qPCR. Data are average ± SE (*n* = 3). (**C**) Protein levels of GATA2, GATA3, AR, and FoxA1 in prostatic lobes as determined by Western blot analysis. Because Western samples for these four transcription factors were from the same batch of prostate tissue lysates, one beta-Actin Western result was shown here to indicate an equal sample loading.

Murine prostate contains three major epithelial cell types, columnar luminal epithelial cells, basal epithelial cells on the basement membrane, and rare neuroendocrine cells. We performed immunofluorescence staining to investigate the expression of GATA2 and GATA3 proteins in epithelial cells in different prostatic lobes. As shown in Figure 1A, GATA2 and GATA3 proteins were predominantly expressed in the nuclei of luminal epithelial cells in AP and DLP (Figure 1A, top and middle panels). Surprisingly, GATA2 or GATA3 is undetectable in the VP by immunofluorescence staining (Figure 1A, bottom two panels). As a control, the basal cell marker p63 protein was specifically expressed in basal cells in all the lobes (Figure 1A). To confirm this finding, by real-time qPCR, we demonstrated that mRNA levels of GATA2 and GATA3 in AP and DLP were also much higher than that of VP (Figure 1B). Next, we performed Western blot analysis. As shown in Figure 1C, the GATA2 and GATA3 protein levels were much higher in AP and DLP than those in VP, in agreement with immunofluorescence staining and real-time qPCR analysis (Figure 1A and 1B). Our results indicate that GATA2 and GATA3 proteins are predominantly expressed in luminal epithelial cells, but not in basal cells in AP and DLP. Moreover, GATA2 and GATA3 expression levels are surprisingly much lower in the VP than those in AP and DLP, suggesting fundamental differences in transcriptional programs among these prostatic lobes.

### GATA2 and GATA3 expression in GATA KO murine prostate

**Figure 2:**
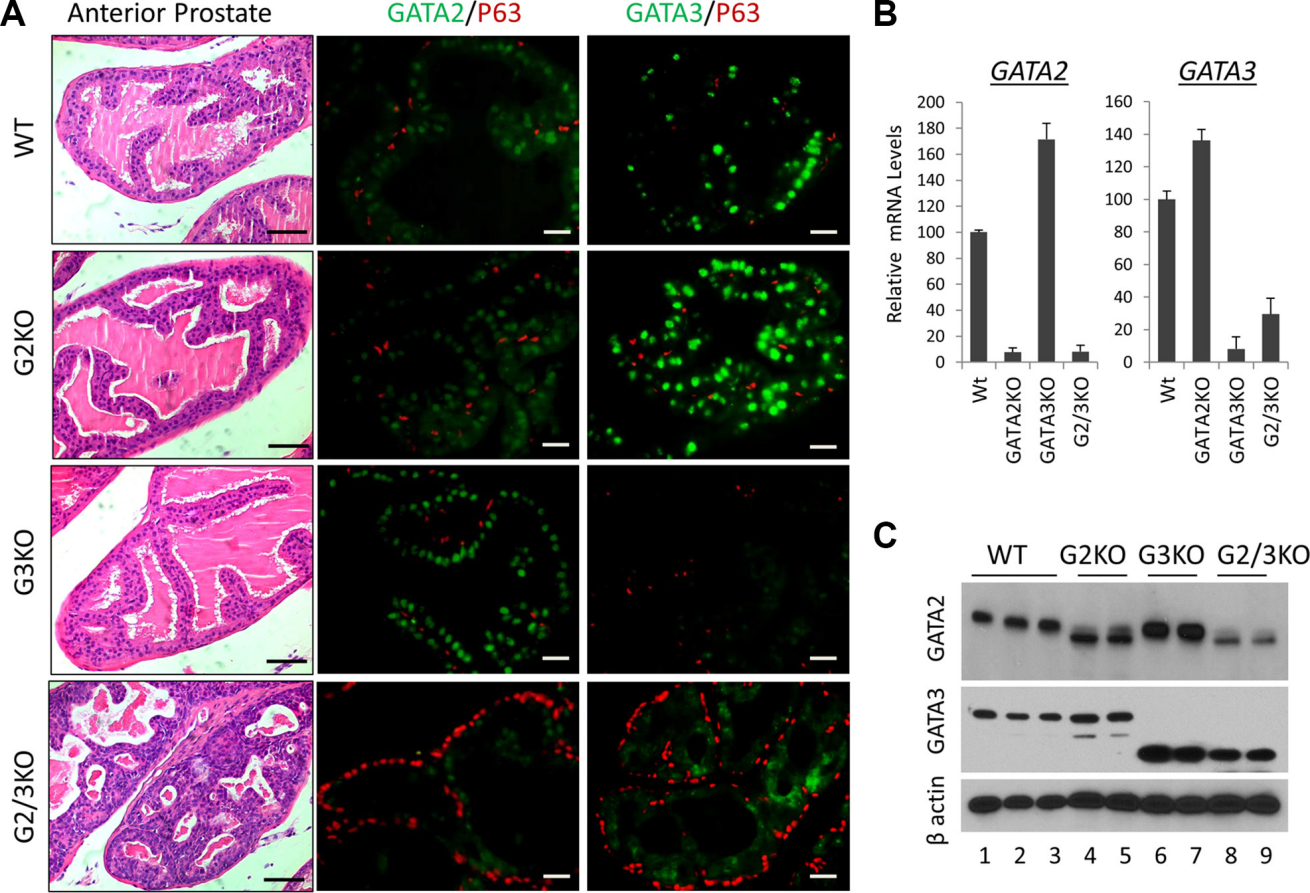
Anterior prostate lobe of the prostate-specific GATA2 and GATA3 KO mice (**A**) left four panels, representative H&E staining of AP lobe of WT and GATA KO mice. G2KO, PBcre;GATA2^fl/fl^. G3KO, PBcre;GATA3^fl/fl^. G2/3KO, PBcre;GATA2^fl/ fl^;GATA3^fl/fl^. The black scale bars, 50 μm. Right eight panels, co-immunofluorescence staining of GATA2 (green), GATA3 (green), and p63 (red). The white scale bars, 20 μm. (**B**) GATA2 and GATA3 mRNA levels in WT and GATA KO AP lobe were determined by qPCR. Data are average ± SE (*n* = 4). (**C**) GATA2 and GATA3 protein levels in GATA KO mouse AP determined by Western blot analysis.

Next to investigate the *in vivo* functions, we generated prostate-specific GATA KO mice by crossing GATA2 and GATA3 conditional knock mice with PB-Cre transgenic mice [28]. The gross histology of the prostate in the GATA2 or GATA3 single KO mice is similar to that of WT mice. However, loss of both GATA2 and GATA3 factors in the double KO resulted in obvious prostatic phenotype. As shown in Supplementary Figure 1B, the relative prostate to body weight of GATA2 and GATA3 double KO (GATA2/3 KO) mice, but not single GATA knockout mice, was significantly less than that of WT mice, indicating that GATA2 and GATA3 are essential for full development of murine prostate. H&E staining showed that anterior prostates of double KO mice resembled PIN III to PIN IV lesions with tufting and micro-papillary patterns, whereas those of single KO mice did not display noticeable histological abnormality (Figure 2A, left four panels). Immunofluorescence staining showed stronger GATA2 staining in GATA3 KO mouse prostate, while GATA3 staining was increased in GATA2 KO mice in the AP (Figure 2A, middle and right panels). This observation was confirmed by real-time qPCR (Figure 2B) and Western blot analysis (Figure 2C), suggesting that GATA2 and GATA3 in the AP are compensating each other at the transcriptional level. We made similar observations in the DLP of GATA KO mice (Supplementary Figure 2). It is worthy to note that anti-GATA3 antibodies for Immunofluorescence staining (rabbit mAb, Cell Signaling) and Western blot (mouse mAb, Santa Cruz) are different. GATA3 immunofluorescence staining mostly disappeared in GATA3 KO mouse (Figure 2A), whereas in Western blot, there were smaller truncated bands in GATA3 single KO and GATA2/3 double KO mice (Figure 2C), indicating two GATA3 antibodies recognize different regions of GATA3 protein. Moreover, in GATA2 knockout mice [29], a truncated GATA2 protein was produced and recognized by anti-GATA2 antibody (rabbit polyclonal Ab, Cell Signaling) in both immunofluorescence staining (Figure 2A) and Western blot analysis (Figure 2C).

### AR expression in GATA KO murine prostate

**Figure 3:**
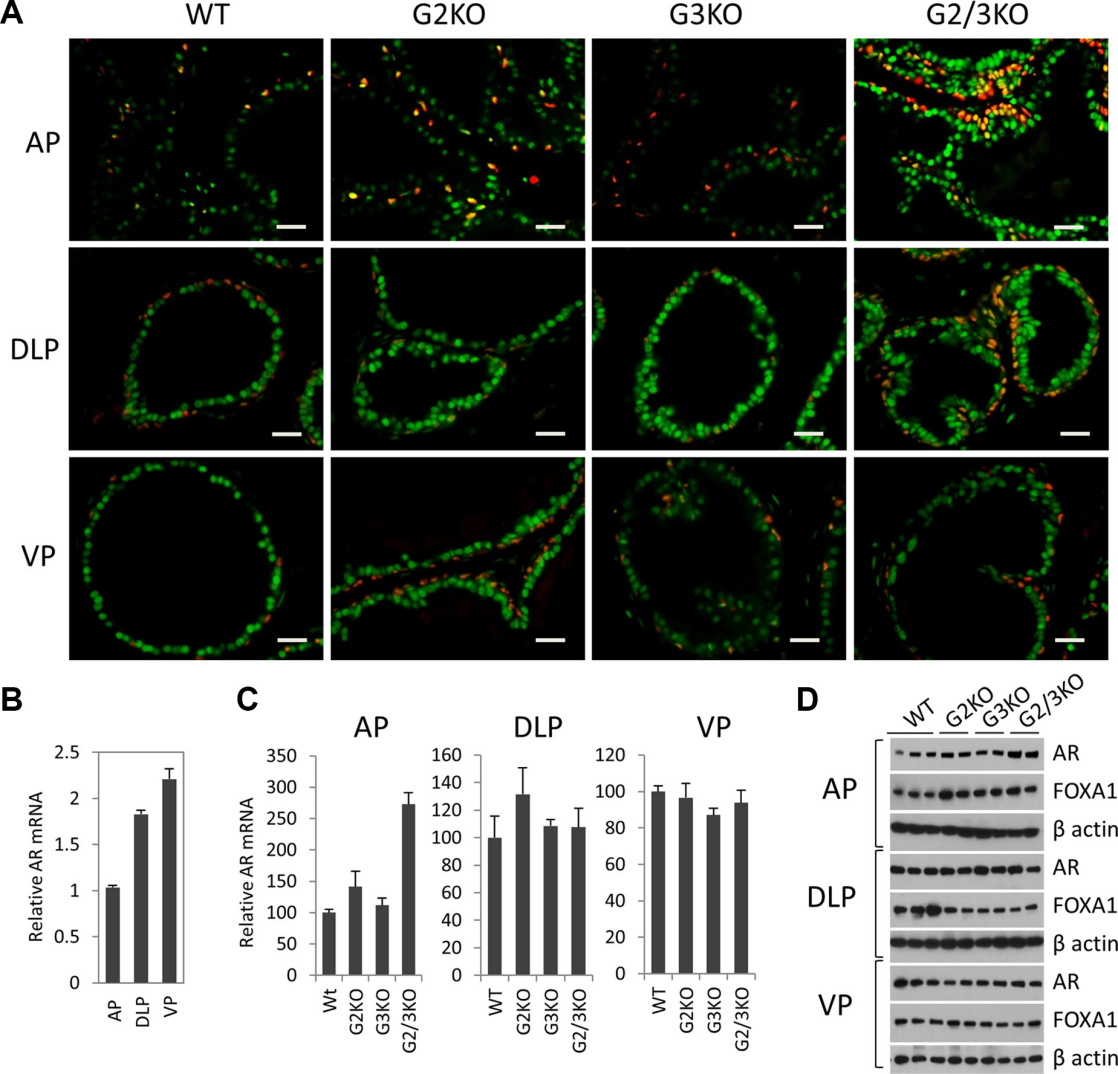
AR expression in luminal and basal epithelial cells in different prostatic lobes in WT and GATA KO mice (**A**) Co-immunofluorescence staining of AR (green) and p63 (red) in prostatic lobes. The scale bars, 20 μm in all panels. (**B**) AR mRNA levels in different prostatic lobes of WT mice were determined by qPCR. Data are average ± SE (*n* = 3). (**C**) AR mRNA levels in different prostatic lobes in GATA KO mice were determined by qPCR. Data are average ± SE (*n* = 4). (**D**) AR and FoxA1 protein levels in different prostatic lobes of WT and GATA KO mice were determined by Western blot analysis.

Androgen signaling has an essential role in the development and normal function of prostate. In cultured prostate cancer cells, GATA2 regulates AR gene expression and also acts as a pioneer factor to cooperate with AR to regulate AR target gene expression [30–32]. We performed the co-immunofluorescence staining to determine the expression of AR in WT and GATA KO prostate. As shown in Figure 3A, AR protein staining was strongly detected in luminal cells and basal epithelial cells in the prostate of WT mice (Figure 3A, left three panels), indicated by the appearance of a yellow color in the basal cells as a result of the merge of green (AR) and red (p63) colors. Interestingly, AR staining appeared to be stronger in DLP and VP than in the AP in WT prostate (Figure 3A, left three panels). This observation is supported by measuring AR expression at mRNA level (Figure 3B) and protein level (Figure 1C), with VP showing the highest level of AR expression.

In the AP of GATA2 and GATA3 single KO mice, the expression levels of AR were similar to that of WT mice. Interestingly, in GATA2 and GATA3 double KO mice, AR expression levels were significantly increased in the AP, as shown by immunofluorescence staining (Figure 3A, top panels), and confirmed by real-time qPCR at mRNA level (Figure 3C) and by Western blot at protein level (Figure 3D). However, in DLP and VP, the expression levels of AR in single KO or double KO mice were similar to that of WT prostate, shown by immunofluorescence staining (Figure 3A, middle and bottom panels) and Western blot at protein level (Figure 3D), and real-time qPCR at mRNA level (Figure 3C). Our results indicate that in adult murine prostate, GATA factors are not required for AR expression. Instead, in the AP, loss of GATA2 and GATA3 increased AR expression, suggesting a negative regulatory mechanism.

### FOXA1 expression in WT and GATA KO murine prostate

**Figure 4:**
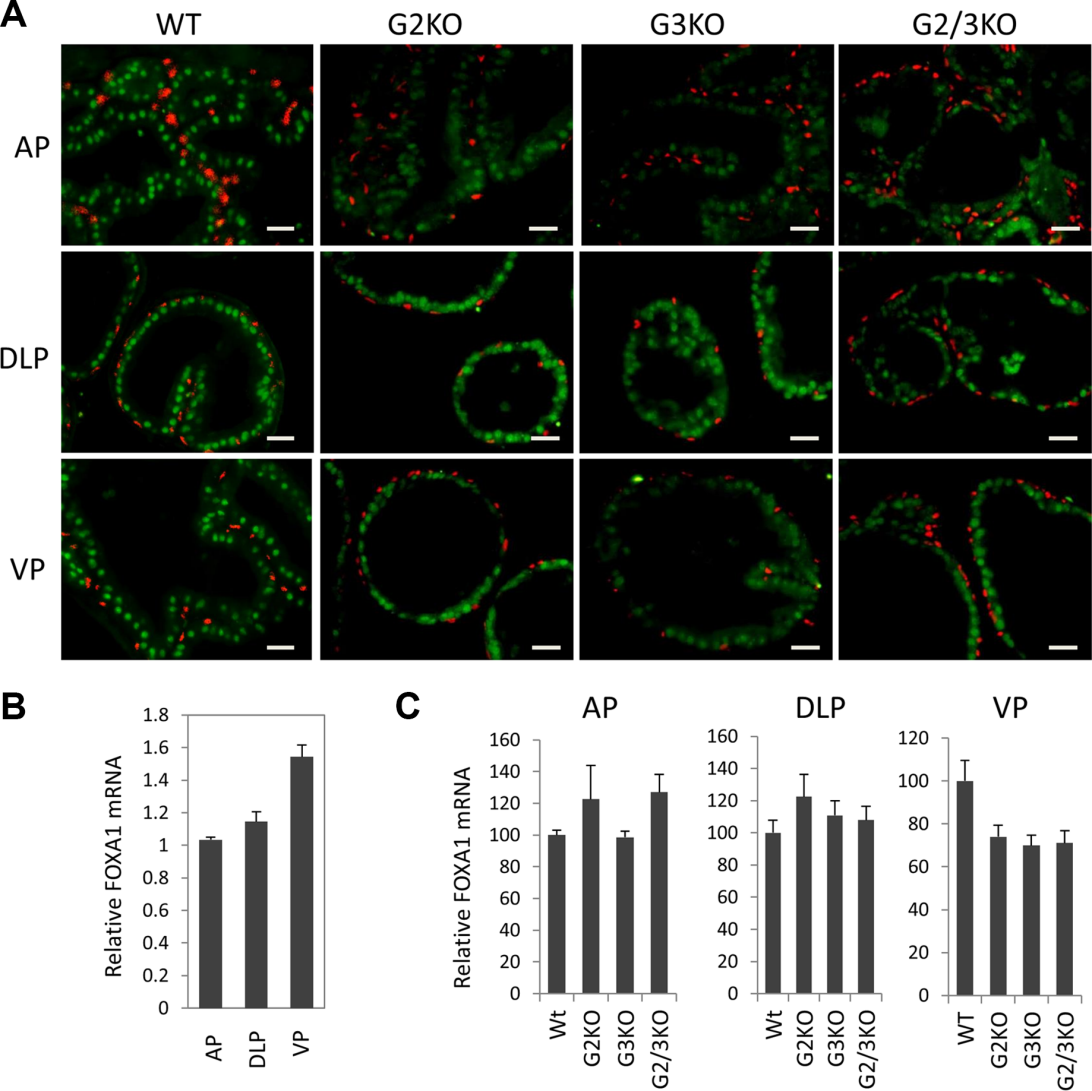
FoxA1 expression in different prostatic lobes of WT and GATA knockout mice (**A**) FoxA1 (green) and p63 (red) co-immunofluorescence staining. The scale bars, 20 μm in all panels. (**B**) FoxA1 mRNA levels in different prostatic lobes of WT mice were determined by qPCR. Data are average ± SE (*n* = 3). (**C**) FoxA1 mRNA levels in different prostatic lobes of WT and GATA KO mice were determined by qPCR. Data are average ± SE (*n* = 4).

FoxA1 is a potent pioneer factor and is crucial for estrogen receptor (ER)-mediated transcription in breast cancer cells [9, 33, 34]. FoxA1 determines AR genomic binding in cultured prostate cancer cells [31, 35, 36] and is frequently mutated in human prostate cancer [37, 38]. *In vivo*, FoxA1 is required for the maintenance of the differentiation status of the prostate epithelial cells in adult murine prostate [13]. Therefore it is important to understand the expression of FoxA1 in WT and GATA KO prostate. By immunofluorescence staining, FOXA1 was exclusively detected in luminal epithelial cells of normal prostate, but not in basal cells (Figure 4A, left three panels). Unlike AR and GATA factors, FOXA1 was expressed at relatively similar levels in different lobes of normal prostate at protein level (Figure 4A and 1C), although FoxA1 mRNA level appeared to be slightly higher in the VP (Figure 4B). Furthermore, loss of GATA2 and/or GATA3 had no dramatic effect on the expression of FOXA1, at both mRNA level (Figure 4C) and protein level (Figure 4A and 3D) in different prostatic lobes. Our results indicate that FOXA1 is expressed at similar levels in different prostatic lobes and its expression is independent of GATA2 and GATA3 status, in support of the hypothesis that FoxA1 functions upstream of AR and GATA factors [31, 39].

### Expansion of p63-positive cells in GATA KO mouse prostate

**Figure 5:**
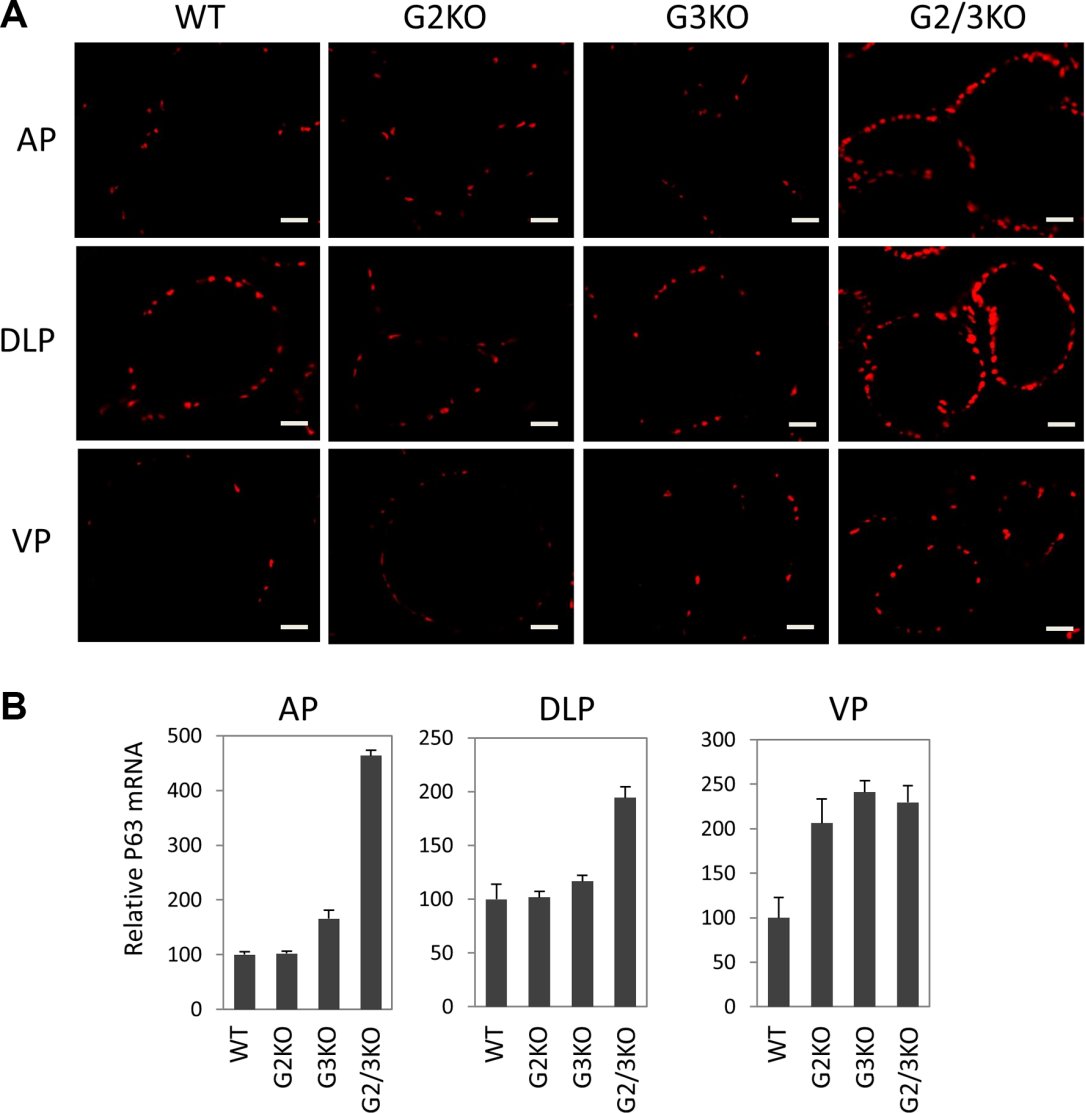
Expansion of p63-positive cells in the prostate of GATA2 and GATA3 double KO murine prostate (**A**) Immunofluorescence staining of p63 (red) in WT and GATA knockout murine prostate, showing a dramatic increase in the number of p63-positve cells in different lobes of GATA2 and GATA3 double knockout mice. The scale bars, 20 μm in all panels. (**B**) p63 mRNA levels in different lobes in WT and GATA KO mice were measured by qPCR. Data are average ± SE (*n* = 4).

In the prostate, p63 is specifically expressed in basal epithelial cells, which contain progenitor cells that can differentiate into luminal and neuroendocrine cells[40]. Therefore it is important to study the p63-positve basal cells in GATA2 and GATA3 KO prostate when the functions of luminal cells are likely to be impaired. Strikingly, p63 immunofluorescence staining showed that GATA2 and GATA3 KO caused dramatical expansion of p63-positive cells in different prostatic lobes (Figure 5A). This finding was in agreement with increased p63 mRNA levels in GATA KO mice shown by real-time qPCR (Figure 5B).

### Role of GATA2 and GATA3 in AR target gene expression in the prostate

**Figure 6:**
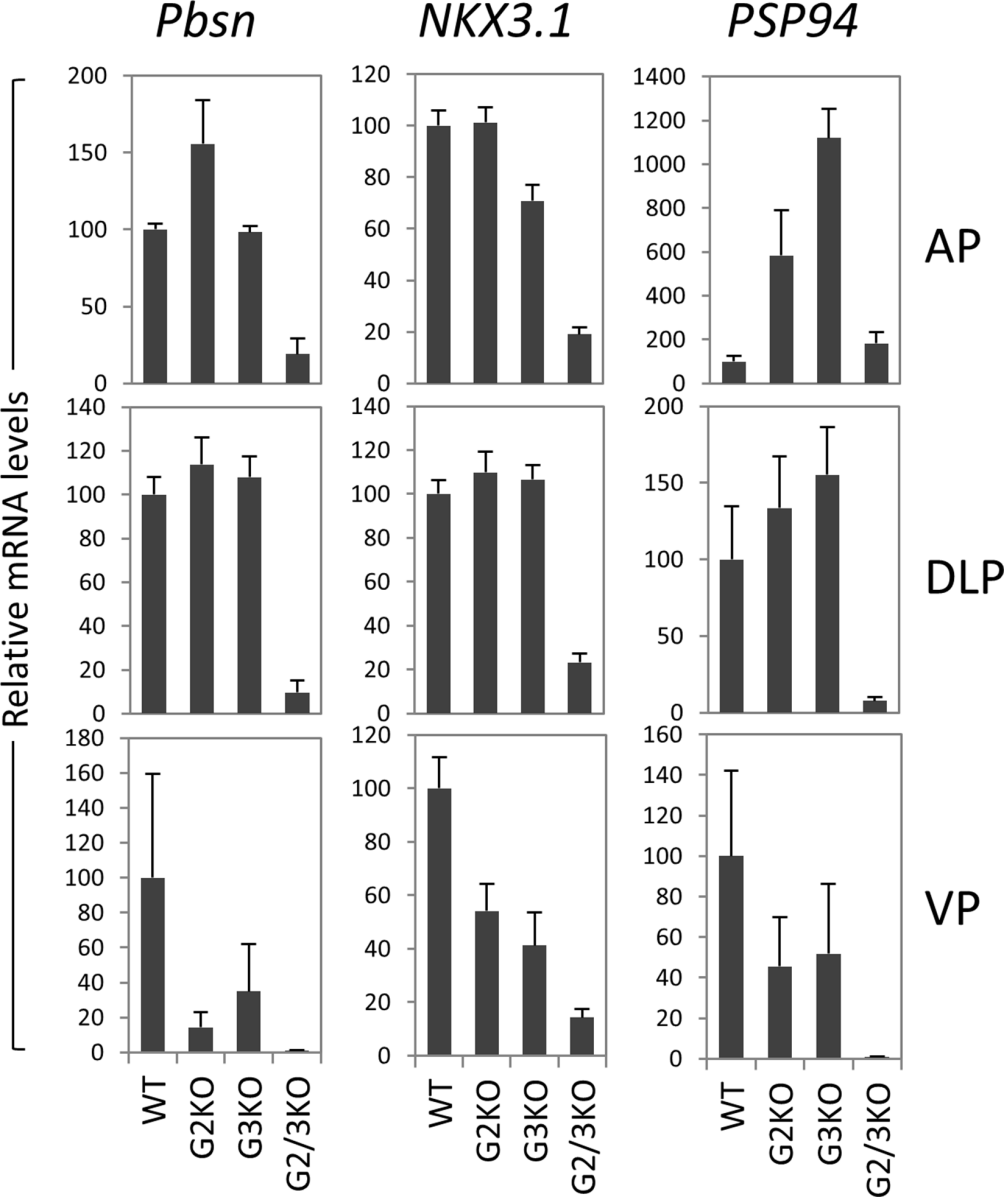
AR target gene expression in the prostate of GATA2 and GATA3 KO mice mRNA levels of Probasin (Pbsn), Nkx3.1, and PSP94 were determined by real-time qPCR in different prostatic lobes of WT and GATA knockout mice. Data are average ± SE (*n* = 4).

In cultured prostate cancer cells, GATA2 is essential for the expression of a subset of AR target genes such as PSA and NKX3.1. We investigated the effect of loss of GATA2 and/or GATA3 on the *in vivo* expression of AR target genes. As shown in Figure 6, in the AP, probasin mRNA levels were dramatically reduced in GATA2 and GATA3 double knock mice, but not in GATA2 or GATA3 single KO mice. NKX3.1 mRNA levels were also reduced in GATA2 and GATA3 double KO mice, suggesting that GATA factors coordinate with AR to regulate Pbsn and Nkx3.1 gene transcription in the AP lobe. In the DLP, GATA2 and GATA3 were also required for the optimal Pbsn and NKX3.1 expression (Figure 6). However not all androgen target genes are regulated by GATA factors in the same manner. The androgen-regulated prostatic secretory protein 94 (PSP94) is one of three most abundant proteins secreted by the human prostate gland and is involved in the regulation of immune response in the female reproductive tract. In mice, PSP94 expression is high in VP and DLP, but low in the AP [41], in agreement with our observation (Supplementary Figure 3). Surprisingly, as shown in Figure 6, in the AP, knockout of GATA2 or GATA3 alone dramatically increased PSP94 mRNA levels, suggesting that GATA2 or GATA3 has suppressive activity on PSP94 expression in the AP. However, knockout of both GATA2 and GATA3 did not further increase PSP94 expression in the AP (Figure 6). In contrast, in DLP and VP, single knockout of GATA2 or GAT3 had minimal effect on PSP94 mRNA expression, whereas knockout of both GATA2 and GATA3 dramatically reduced the PSP94 mRNA levels, indicating that GATA factors regulate PSP94 gene transcription through distinct mechanisms in different prostatic lobes. Taken together, our results demonstrate an *in vivo* role for the GATA factors in the regulation of AR target gene expression in the murine prostate and reveal an unexpected diversity in the regulatory mechanisms.

## DISCUSSION

### Transcriptional regulation of prostatic epithelial cell functions

The adult prostate epithelial cells are composed of three main cell lineages: luminal epithelial cells that express keratin K8, K19, and secrete prostate-specific antigen (PSA); basal epithelial cells that express p63, k5 and k14; and rare neuroendocrine cells that express chromogranin A and synaptophysin. Prostatic basal epithelial cells express DeltaNp63, the predominant isoform of p63 in the prostate. p63-positive basal cells are the stem cells of developing prostate epithelium that can differentiate into luminal and neuroendocrine epithelial cells [40, 42, 43]. p63-null mice do not develop the prostate [44]. In contrast, the secretory luminal epithelial cells are well differentiated, and express genes such as PSA in human prostate and probasin in murine prostate. The role of AR in luminal cell differentiation and function is well established. As shown in human prostate adenocarcinoma cancer cells, AR directly binds to PSA promoter and enhancer regions and up-regulate PSA gene transcription. In adult murine prostate, probasin is abundantly expressed in luminal cells, but not in basal cells. Knockout of GATA2 and GATA3 dramatically reduced probasin mRNA levels, indicating that GATA factors function as key pioneer/coordinating factors for AR to drive Pbsn expression.

Shown by immunofluorescence staining with a rabbit monoclonal antibody, AR protein is highly expressed in luminal epithelial cells, but also in basal epithelial cells, which is in agreement with a previous report in which basal cells and luminal cells were purified and analyzed [45]. It has been established that AR transactivation of target genes in cultured prostate cancer cells requires pioneer factors, such as FoxA1 and GATA2 [30]. Our findings are that FoxA1, GATA2, and GATA3 proteins are expressed predominantly in luminal epithelial cells, but not in basal cells, implying that AR in basal cells might not be functional, and therefore could not drive the expression of genes such as PSA (human), probasin (mouse), and Nkx3.1. Nevertheless, staining shows that AR protein in basal epithelial cells clearly localizes in the nuclei. It is thus also possible that AR in basal epithelial cells has FoxA1- and GATA-independent transcriptional activity, or even transcription-independent function.

Our results show that knockout of both GATA2 and GATA3 causes dramatic expansion of p63-positive basal cells. Luminal epithelial cells are differentiated from p63-positive basal cells [40], and these two cell types likely have reached equilibrium in adult prostate. Disruption of luminal cells by GATA2 and GATA3 double KO would stimulate proliferation of p63-positive basal cells by an unknown feedback mechanism and result in p63-positive cell expansion. However, knockout of GATA2 and GATA3 did not convert luminal cells into p63-positive basal cells as shown by immunofluorescence staining (data not shown), indicating other key transcription factors such as AR and FoxA1 might be able to sustain the differentiation status of luminal cells.

### GATA3 function in the prostate

GATA factors are essential for steroid receptor-mediated transcription. In the murine mammary gland, GATA3 is the only GATA member that expressed in the differentiated luminal epithelial cells and is critical for maintenance of the differentiation status [20]. In cultured breast cancer cells, GATA3 and ERα positively regulate each other's expression, forming a positive regulatory loop [46]. On the other hand, GATA2 is the only GATA member expressed in the uterus [47]. GATA2 is a progesterone receptor (PR) target gene [48] and cooperates with PR to regulate PR target gene expression [49, 50]. Therefore it is not surprising that GATA factors also play an important role in AR signaling in the prostate. Strikingly, unlike GATA3 alone in the mammary gland or GATA2 alone in the uterus, both GATA2 and GATA3 are expressed in the murine and human prostate, suggesting a coordinated transcriptional program regulated by two GATA factors in the prostate.

However, GATA3 is not expressed in any human prostate cancer cell lines at mRNA [51] or protein levels (unpublished data). During human prostate cancer development, GATA3 mRNA levels are found to be significantly reduced in primary prostate tumors [30, 52], in contrast to GATA2 whose mRNA levels are unchanged in primary prostate tumors and moderately increased in metastatic prostate tumors [30, 52]. These results suggest that GATA3 might act as a tumor suppressor and is thus lost at the early stage during prostate cancer development. Importantly during breast cancer development, GATA3 indeed has a tumor suppressor function. Loss of GATA3 expression marks the progression from adenoma to early carcinoma in multiple mouse models of breast cancer development [53]. GATA3 is one of a few most frequently mutated genes in human breast tumors, and the majority of GATA3 mutations result in frameshift and loss of normal GATA3 function. GATA3 also suppresses metastasis by regulating miR-29b expression [54]. Therefore, it is tempting to speculate that in the prostate, GATA3 also has a tumor suppressor activity and loss of GATA3 is required for prostate tumorigenesis. The tumor suppressor function of GATA3 is in agreement with the observation that, as shown by the H&E staining, the AP and DLP of GATA2/3 double KO mice resembled PIN III to PIN IV lesions with tufting and micro-papillary patterns (Figure 2A and Supplementary Figure 2A). However, in the GATA2/3 double KO mice, the AR transcriptional activity was significantly reduced as measured by the expression levels of AR target genes (Figure 6), suggesting that the phenotype of GATA2/3 double KO mice might not be the same as the typical PIN lesions observed in PTEN knockout mice [55]. The mechanism of how these tufting structures were formed in the GATA2/3 KO mice remains to be investigated.

### Lack of GATA2 and GATA3 expression in ventral prostatic lobe

One striking finding from our study is the lack of GATA2 and GATA3 expression in ventral prostatic lobe, shown by immunofluorescence staining, Western blot analysis, and real-time qPCR. In contrast, two other key transcription factors, FoxA1 and AR, are similarly expressed in all the lobes. The differential gene expression patterns between AP, DLP, and the VP were previously investigated by an expression microarray analysis [5]. We downloaded their microarray data, and confirmed that the microarray-based mRNA levels of both GATA2 and GATA3 are three times lower in the VP than those of AP and DLP, which is in agreement with our findings. Moreover, lack of GATA2 and GATA3 expression in VP is not compensated by increased expression of other GATA family members based on the published microarray analysis [5] and our own real-time qPCR analysis (data not shown). Our results show that GATA2 and GATA3 are critical for the expression of a subset of AR target genes, including probasin. This finding is in agreement with much lower expression levels of probasin in the VP than that of AP and DLP, given the essential role of GATA2 and GATA3 in AR-driven probasin expression. Therefore the lack of GATA factor expression suggests a fundamental difference between the VP and other murine prostatic lobes.

Finally, human prostate is divided into functional zones, the central zone, transition zone, and peripheral zone, which are implicated in different diseases. Prostate cancer arise mainly in the peripheral zone, while benign prostatic hyperplasia (BPH) occurs only in the transition zone [1]. It remains to be determined whether genetic difference, such as differential expression of GATA factors, could have caused a functional difference in different human prostate zones. Better understanding of the genetic and molecular differences between human prostate zones will shed light on the pathophysiology of human prostate diseases.

## MATERIALS AND METHODS

### Genetically engineered mouse models and genotyping

All mouse experiments were performed in accordance with IACUC-approved procedures (Baylor College of Medicine, Houston, TX). GATA2 conditional KO mouse model (GATA2^fl/fl^) was previously reported [29]. The mice were obtained from the Mutant Mouse Resource & Research Centers (MMRRC) and are on a mixed C57BL/6/129 genetic background. GATA3 conditional KO mouse has been previously described [56]. ARR2PB-Cre (PBCre) transgenic mouse model was previously reported [28]. The PCR primers for genotyping are as follows: for GATA2 conditional KO mice, 5′-TCC GTG GGA CCT GTT TCC TTA-3′ and 5′-TCG TCT GAC AAT TTG CAC AAC-3′. The sizes of PCR products are 346 bp for wild type (WT) allele and 400 bp for floxed allele. For GATA3 conditional KO mice, 5′-TCA GGG CAC TAA GGG TTG TTA ACT T-3′; and 5′-GAA TTC CAT CCA TGA GAC ACA CAA-3′. The PCR products are 150 bp for WT allele and 220 bp for floxed allele. For PBCre transgenic mouse: 5′-GGG TCG ATG CAA CGA GTG AT-3′; and 5′-CCA CCG TCA GTA CGT GAG AT-3′. The PCR product for PBCre transgene is ~400 bp. Because the mice are on a C57BL/6/129 mixed background, we first bred the mice to obtain GATA2^fl/+^;GATA3^fl/+^ and PBCre;GATA2^fl/+^;GATA3^fl/+^. Then we crossed GATA2^fl/+^;GATA3^fl/+^ with PBCre;GATA2^fl/+^;GATA3^fl/+^ to obtain the following six types of compound mice: (1) PBCre;GATA2^fl/fl^, (2) GATA2^fl/fl^, (3) PBCre;GATA3^fl/fl^, (4) GATA3^fl/fl^, (5) PBCre;GATA2^fl/fl^;GATA3^fl/fl^, and (6) GATA2^fl/fl^;GATA3^fl/fl^. Subsequently, GATA2^fl/fl^ was bred to PBCre;GATA2^fl/fl^ to obtain more GATA2 KO mice; GATA3^fl/fl^ was bred to PBCre;GATA3^fl/fl^ to obtain more GATA3 KO mice; and GATA2^fl/fl^;GATA3^fl/fl^ was bred to PBCre;GATA2^fl/fl^;GATA3^fl/fl^ to obtain more GATA2 and GATA3 double KO mice. GATA2^fl/fl^, GATA3^fl/ fl^, and GATA2^fl/fl^;GATA3^fl/fl^ are considered as WT. For comparison, littermates were used whenever they were available.

### Harvest of fresh prostate tissues

The body weights of 2–4 month old mice were measured before being euthanized. The prostate was dissected in cold PBS buffer under a dissecting microscope. After measuring the total weight, the whole prostate was separated into individual lobes including AP, DLP, and VP [4]. Half sample of each lobe was flash frozen in liquid nitrogen for later RNA and protein extraction; the other half lobe was fixed in 10% formalin for immunohistochemistry analysis. The relative prostate weight was determined as percentage of the total body weight.

### Quantitative real-time PCR

Total RNA was isolated from different prostatic lobes using Trizol Reagent (Invitrogen, Carlsbad, CA). RNA concentrations were determined using a spectrophotometer (GE NanoVue, USA) and were diluted to 1 μg/μl in RNase-free water (Ambion). Real time PCR was conducted by Applied Biosystems Fast One system, SensiFAST SYBR No-ROX Kit (Bioline) was used, using 1–4 μg of RNA as template. Beta-actin (ACTB) was used as an internal control. qPCR primer sequences are as follows: mACTB, 5′-aaggccaaccgtgaaaagat-3′ and 5′-gtggtacgaccagaggcatac-3′. mGATA1, 5′-tggcacaggac agtccaag-3′ and 5′-gggcaagggttctgaggt-3′. mGATA2, 5′-cacaagatgaatggacagaacc-3′ and 5′-acaggtgcccgctcttct-3′. mGATA3, 5′-ttatcaagcccaagcgaag-3′ and 5′-tggtggtggtctg acagttc-3′. mGATA4, 5′-ggaagacaccccaatctcg-3′ and 5′-catg gccccacaattgac-3′. mGATA5, 5′-ccttcgacagcagcatcc-3′ and 5′-tcctccaagaagtcaggtacg-3′. mGATA6, 5′-ggtctctacag caagatgaatgg-3′ and 5′-tggcacaggacagtccaag-3′. mAR, 5′-aatgagtaccgcatgcacaa-3′ and 5′-cccatccactggaataatgc-3′. mFoxA1, 5′-gaacagctactacgcggaca-3′ and 5′-cggagttcatgt tgctgaca-3′. mNKX3.1, 5′-cgactgaacccgagtctgat-3′ and 5′-aatcacctgagtgtgagagaagg-3′. mPbsn, 5′-ctcctgctcacactg cat-3′ and 5′-caaggcccgtcaatcttc-3′. mPSP94, 5′-tttccaaatcaa atgtctgatga-3′ and 5′-gggagtgttaaggaaatgcttg-3′. mp63, 5′-cagccatgcccagtatgtag-3′ and 5′-ttgtgaattcagtgccaacc-3′. mCK5, 5′-ccttcgaaacaccaagcac-6′ and 5′-gttctggaggtt ggcacact-3′. mCK8, 5′-agttcgcctccttcattgac-3′ and 5′-gctgc aacaggctccact-3′. It is worthy to note that for GATA2 and GATA3 qPCR assays, one primer is located in the exon being deleted in the knockout mice. As a result, the GATA2 or GATA3 qPCR can only detect wild type, but not mutant mRNA.

### Hematoxylin and eosin (H&E) and immunofluorescence staining

After overnight fixation in 10% formalin, the prostate tissues were dehydrated and paraffin embedded. Five micrometer sections were cut. H&E staining was performed as previously described [57]. For IF staining, after removing the paraffin wax, the sections were rehydrated, and antigen retrieval was performed by boiling the sections in 0.1 M citrate buffer (pH 6.0) for 20 min followed by cooling at room temperature for 30 min. Non-specific binding was blocked with 1% Blocking Reagent in PBS for 1 hr at room temperature. The sections were incubated with primary antibodies diluted with 1% Blocking Reagent buffer overnight at 4°C. After three rinses with PBS buffer, the sections were then incubated with Alexa Fluor^®^ conjugated secondary antibodies for 1 hr at room temperature. After rinsing three times with PBS, the sections were mounted with SlowFade® Gold Antifade Reagent with DAPI. Images were captured through a Zeiss Axioskop-2 microscope (Carl Zeiss, LLC, Thornwood, NY). When we acquired the fluorescence images, the exposure time was fixed to allow comparison of staining intensities between different sections.

### Western blot analysis

Prostatic lobe tissue was homogenized and protein extracted in tissue lysis RIPA buffer (10 mM TrisCl, pH8.0, 1 mM EDTA, 0.5 mM EGTA, 1% Triton X-100, 0.1% sodium deoxycholate, 0.1% SDS, 140 mM NaCl, 1 mM PMSF). After measuring the concentrations with Bio-Rad Protein Assay Reagent, protein lysates were diluted to 3μg/μl with lysis buffer and denatured by boiling 10 min with 1X Laemmli buffer. Thirty μg proteins were separated on 4–15% SDS-polyacrylamide gradient gels followed by transfer of the proteins to polyvinylidene difluoride (PVDF) or nitrocellulose membranes at 4°C. The membranes were blocked in 5% milk in TBST (10 mM TrisCl, pH 8.0, 150 mM NaCl, 0.05% Tween) for 1 hr at room temperature. Then the membranes were incubated with primary antibodies diluted in 5% milk in TBST at 4°C overnight with rotation. After washing three times with TBST buffer, the blots were incubated with HRP-conjugated secondary antibodies for 1 hr at room temperature. The blots were washed at least three times with TBST buffer. Immunoreactive bands were visualized using chemiluminescence (SuperSignal West Dura Extended Duration substrate; Piece, Rockford, IL) and x-ray film.

### Commercial antibodies

(1) Anti-AR, rabbit monoclonal Ab (mAb), Abcam, cat# ab133273 for immunofluorescence staining and Western. (2) Anti-FoxA1, rabbit mAb, Abcam, cat# ab99892 for immunofluorescence staining and Western blot. (3) Anti-GATA2, rabbit polyclonal Ab, Cell Signaling, cat#4595 for immunofluorescence staining and Western blot. (4) Anti-GATA3, rabbit mAb, Cell Signaling, cat# 5852 for immunofluorescence staining; mouse monoclonal Ab, Santa Cruz, cat# sc-269 for Western blot. (5) Anti-p63, mouse mAb, Santa Cruz, cat# sc-8431. (6) Anti-beta Actin, mouse mAb, Sigma, cat# A5316. (7) Secondary antibodies for immunofluorescence staining were purchased from Invitrogen: anti-mouse, Alexa Fluor 568, A10037; anti-mouse, Alexa Fluor 647, A21236; anti-mouse, Alexa Fluor 594, R37115; anti-rabbit, Alexa Fluor 488, R37118; anti-rabbit, Alexa Fluor 568, A11011.

### Statistical analysis

The statistical significance was analyzed by two-tailed Student's *t*-test. *P*-values < 0.05 were considered statistically significant.

## SUPPLEMENTARY MATERIALS


