# Supplementary material for: The essential role of GATA transcription factors in adult murine prostate
Source: Oncotarget. 2016 Jun 25;7(30):47891–903. doi: 10.18632/oncotarget.10294 (PMC5216986; doi:10.18632/oncotarget.10294)
Supplement: Supplementary file 1 [file oncotarget-07-47891-s001.pdf]

The essential role of GATA transcription factors in adult murine prostate

Supplementary Materials

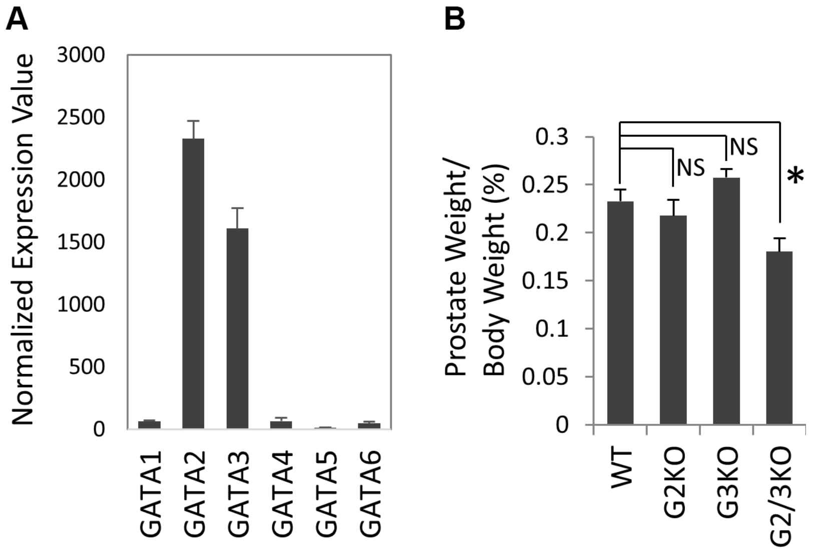

Supplementary Figure S1: (**A**) Microarray-based mRNA expression levels of six GATA factors in isolated total prostate epithelial cells. Total epithelial cells were purified as described [58]. (**B**) Effect of GATA knockout on the relative prostate weight. WT, *n* = 14; GATA2 KO, *n* = 10; GATA3 KO, *n* = 7; GATA2/3 double KO, *n* = 11. **p* < 0.05, two-tailed *t*-test. NS, not significant.

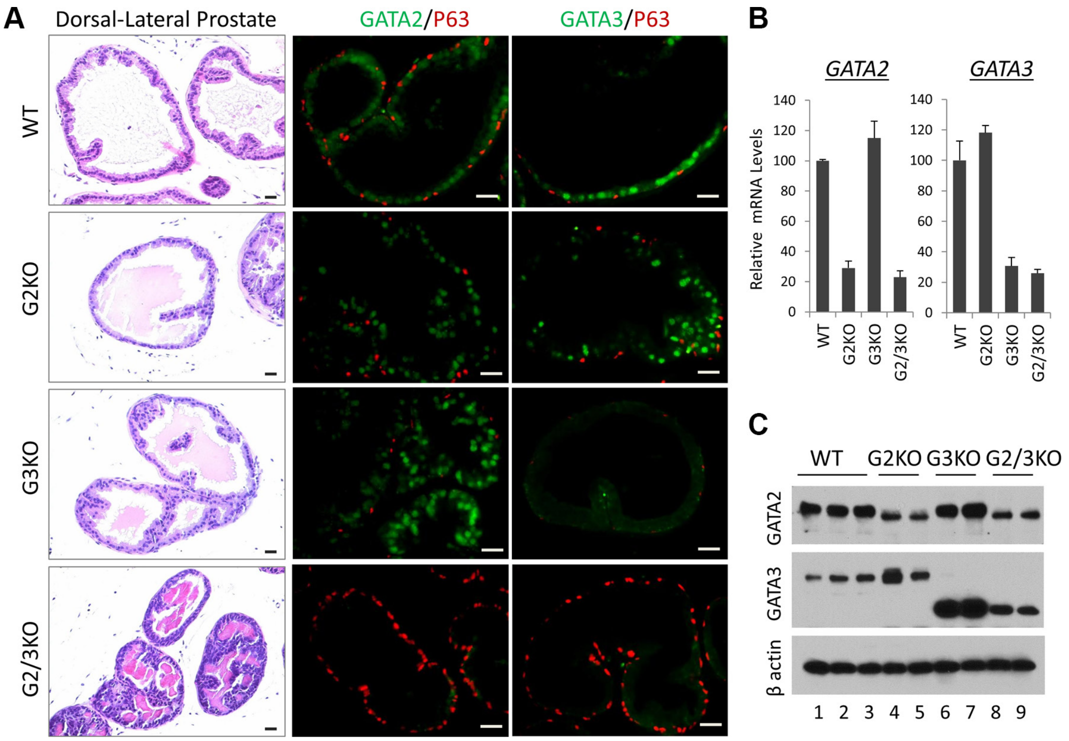

Supplementary Figure S2: DLP phenotype of prostate-specific GATA2 and GATA3 KO mice. (**A**) Left four panels, representative H&E staining of DLP lobe of WT and GATA KO mice. The black scale bars, 20 µm. Right eight panels, co-immunofluorescence staining of GATA2 (green), GATA3 (green), and p63 (red). The white scale bars, 20 µm. (**B**) GATA2 and GATA3 mRNA levels in GATA KO mouse DLP were measured by qPCR. Data are average ± SE (*n* =4). (D) GATA2 and GATA3 protein levels in GATA KO mouse DLP were determined by Western blot analysis.

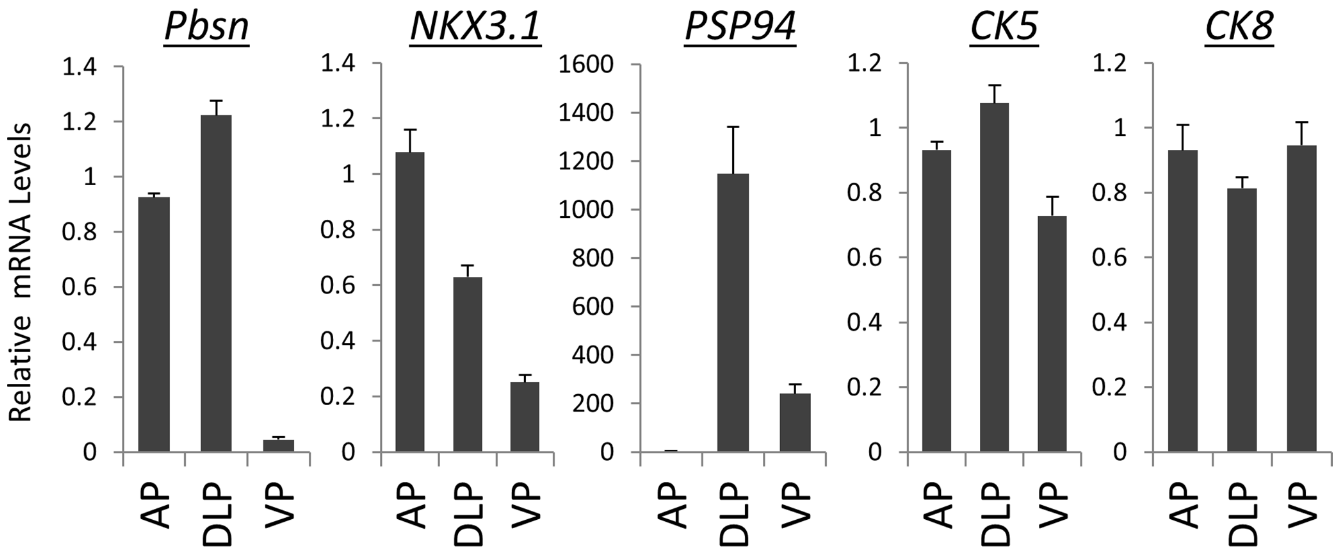

Supplementary Figure S3: mRNA levels of AR target genes (Pbsn, NKX3.1, PSP94) and epithelial cell marker genes (CK5 and CK8) in different prostate lobes of WT mice were determined by qPCR. Data are average ± SE (*n* = 3).
